# Quasi-perfect vortices generated by Pancharatnam-Berry phase metasurfaces for optical spanners and OAM communication

**DOI:** 10.1038/s41598-022-05017-0

**Published:** 2022-01-20

**Authors:** Zhiyuan Xiang, Zhe Shen, Yaochun Shen

**Affiliations:** 1 School of Electronic and Optical Engineering, Nanjing University of Science and Technology, Nanjing, 210094 China; 2 Department of Electrical Engineering & Electronics, University of Liverpool, Liverpool, L69 3GJ UK

**Keywords:** Metamaterials, Optical manipulation and tweezers, Micro-optics, Micro-optics, Fibre optics and optical communications

## Abstract

Optical vortex (OV) can be used in the fields of optical manipulation and optical communication because of its inherent orbital angular momentum (OAM). The size of the OV ring increases with the correlated topological charge (TC), making the OV with large TC not suitable for optical rotation and short-distance communication. Perfect vortex (PV) has attracted much attention due to that its optical transmission profile is almost independent of TC. In this manuscript, we proposed a method to generate quasi- perfect vortices (Q-PVs) by Pancharatnam–Berry (PB) phase metasurfaces, the so-called Q-PV can be regarded as an annularly focused optical vortex whose focal ring in the focal plane has an angular phase gradient. It has a similar property to PV in that its light profile hardly changes with TC in the focal plane. We demonstrated that the Q-PV can be used for optical spanners that particles are trapped and rotated on the specific orbit. Non-coaxial and coaxial Q-PV arrays were further generated for OAM communication applications. We believe that the proposed Q-PVs has potential applications in optical manipulation and optical communication.

## Introduction

Optical vortex (OV) as a kind of Laguerre-Gaussian (LG) mode beam with spiral phase and orbital angular momentum (OAM), has main applications in the fields of particle trapping and manipulation^1–3^ and optical communication^4–6^. OV has singularity with a donut shape, leading to destructive interference at the central light field. Due to the oblique feature of the Poynting vector in the OAM field, the trapped particles will rotate along the OAM orbit field^7,8^. In the past several years, OV has been developed as an optical spanner and received wide attention^2,3,9–11^. Various kinds of optical spanners such as focused optical spanners^12^, plasmonic spanners^13^ have been developed. The complex amplitude of OV has Hilbert factor exp(*ilθ*), where $l$ is the number of topological charge (TC), and $\theta$ is the azimuth angle^14^. TC is the key parameter of OV, and it is proportional to the OAM and the radius of light field. Because of the changeability of OAM, OV has a wide range applications in the field of optical communication, such as quantum information coding^15,16^, wavelength division multiplexing(WDM)^17^, Dammann grating splitting^18,19^. However, the ring radius of the vortex intensity profile strongly depends on the TC, thus limiting its application on fiber coupling and multiplexing. OAM sources with changeable TCs as well as fixed rings are highly desirable for optical manipulation and communications^20^.

Perfect vortex (PV), whose ring profile and divergence angle keep the same size under different TCs^21–23^, could provide a perfect solution. Traditional PVs were generated by the Fourier transform of a Bessel-Gaussian beam^24^ by using several bulky optical elements including conical lens, spiral phase plate or spatial light modulator (SLM), and lens^22,25,26^. Alternatively, PV has also been generated by using conical lens to generate annular beam and SLM to mark vortex phase, and it has been used to achieve fixed orbit OAM manipulation in the tight focal field^25^. In 2014, a similar beam was generated by SLM imprinting the phases of the diffraction ring lens and spiral phase plate^27^. However, the generation of these beams requires complex and bulky optical elements and precise light path adjustment.

Recently, the study of metasurfaces makes optical devices tend to be flattened and integrated. Dielectric metasurfaces consist of nanofins in subwavelength scale, providing a powerful and efficient platform for light field regulation in cases of amplitude, phase, and polarization^28,29^. Pancharatnam-Berry (PB) phase optical elements use the idea of geometric phase with polarization transmission, which recently has been used in the applications of OV generation^30,31^ and OV spanners^31,32^. There have appeared some excellent works for PV generation by using PB phase metalens. In 2017, Liu proposed the generation of PV by the combination of three metalenses with convex lens phase, Bessel phase, and vortex phase^20^. In 2018, Yang combined these phases on one single plasmonic metasurface based on PB phase to obtain a 3D broadband PV^33^.

In this manuscript, the dielectric metasurfaces based on PB phase were employed to combine the annular focusing phase and OV phase to generate quasi- perfect vortex (Q-PV). The Q-PV beam in our work can be understood by adding angular phase gradient to the focal ring in the focal plane formed by the metasurfaces. Different from the previous quasi-PV based on the Fourier transform of high-order quasi-Bessel beams^34^, our Q-PV beam emphasizes a PV-like effect, which has completely difference in the generation mechanism. It is expected that the generated Q-PVs with different TCs have fixed light field profiles in the focal planes. The generated Q-PV has technically eliminated the Bessel phase, which generally exists in traditional PV due to the Bessel series expansion^20,33^. With the generated Q-PV, 3D optical trapping can be achieved since the size and position of the focal ring are adjustable, while only 2D trapping can be achieved with traditional PV due to the non-diffraction propagation property. Besides, we can generate a new kind of optical spanner in that particles are rotated on a fixed orbit with different torques. The Q-PV can be used in OAM communication for integrated photonic devices. Since the metasurface can easily form an array, either coaxial or non-coaxial communication can be achieved. All in all, the proposed Q-PV may broaden the applications of optical information coding and storage, photon computing, micromechanics, and lab-on-a-chip.

## Results

### Theoretical description of Q-PV

Our proposed Q-PV is ideally defined by

1 $$E\left(r,\theta ,f\right)=\delta \left(r-{r}_{0}\right)\cdot \mathrm{exp}\left(il\theta \right), \left(l=1, 2, 3\dots \right),$$

where *f* indicates that the Q-PV light field will be formed at the *z* = *f* plane, *r*, *θ* are the polar coordinates in the beam cross-section in the focal plane, *δ*(*) is the Dirac *δ*-function, *l* is the TC and *r*_0_ is the ring radius of the light field. The part after the equal sign keeps exactly the same form as that of PV^23^. It means the Q-PV has the same property with PV in the focal plane that the maximum intensity ring at *r*_0_ has noting with TC. Function *δ*(*) can be expanded based on the principle of generalized Snell's law^34^, and the transmission function of Q-PV can be expressed as

2 $$T\left(r,\theta \right)=\mathrm{exp}\left(ik\left(f-\sqrt{{f}^{2}+{\left(r-{r}_{0}\right)}^{2}}\right)\right)\cdot \mathrm{exp}\left(il\theta \right),$$

where $k=2\pi /\lambda$ is the wave vector at the working wavelength *λ*, *θ* is the azimuth angle, *l* is the number of TC, respectively. Fresnel diffraction integral theory was used to accurately calculate the light field distribution. The field intensity of Q-PV on the *z*-transmission plane can be expressed as

3 $$E\left(r,\rho ,z\right)=\frac{k}{z}{\int }_{0}^{{\rho }_{0}}\mathrm{exp}\left(ik\left(\sqrt{{r}^{2}+{z}^{2}}-\sqrt{{f}^{2}+{\left(r-{r}_{0}\right)}^{2}}\right)\right)\cdot {J}_{l}\left(\frac{k\rho r}{z}\right)rdr,$$

where *ρ* is the integral radius of the aperture, *ρ*_*0*_ is the lens size, and *J*_*l*_(*) is the *l*^th^ Bessel function, which can be considered as the combination of the circular aperture diffraction of the PB phase metasurface and the *l*^th^ OV light.

### The metasurface design

**Figure 1:**
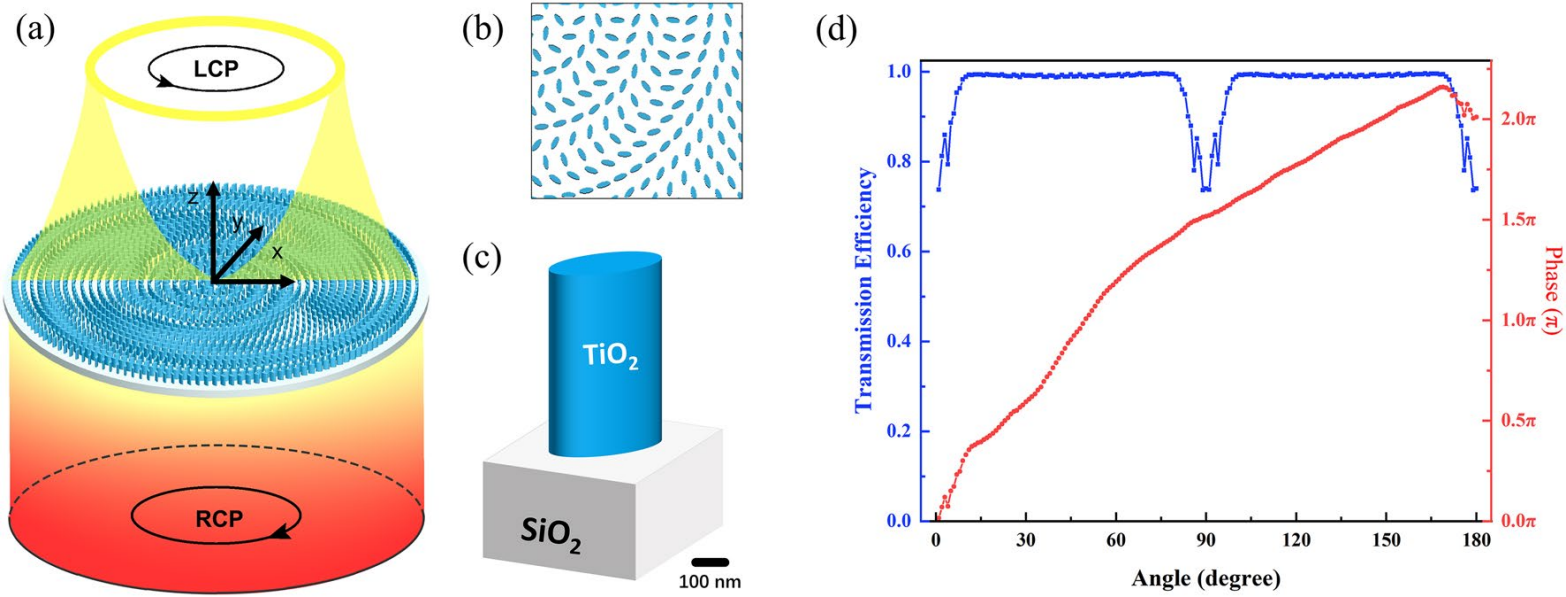
The metasurface design for generating Q-PV. (**a**) The schematic for generating Q-PV by PB phase metasurface. (**b**) The enlarged local part of the PB phase metasurface. (**c**) The structure of each nano-elliptic unit in one period. (**d**) The distribution of the transmission efficiency and the phase delay in the case of the angle between the long axis of the nanopost and the positive half-axis of the *x*-axis are 0 to 180 (degree).

We used an all-dielectric PB phase metasurface consisting of elliptic nanoposts^35^ array on a SiO_2_ substrate, as shown in Fig. 1a. A right-handed circularly polarized (RCP) Gaussian light normally illuminated the SiO_2_ substrate from the bottom up, passing through the nanoposts array and form a Q-PV with left-handed circularly polarized (LCP) state^36^. The beam’s working wavelength was set with 532 nm and the waist radius was twice the radius of the metasurface. To uniformly produce a Q-PV, the nanoposts on the SiO_2_ substrate were arranged in ring shape. According to PB phase principle, the rotation angle of each nanopost should be $\alpha = \varphi /2$. Extract the phase term of Eq. 2 and accept the phase-angle conversion relationship, we obtain the following expressions for the phase and rotation angle distributions, respectively:

4 $$\varphi \left(r,l,\theta \right)=k\left(\left(f-\sqrt{{f}^{2}+{\left(r-{r}_{0}\right)}^{2}}\right)\right)+l\cdot \theta ,$$

5 $$\alpha \left(r,l,\theta \right)=\frac{k}{2}\left(\left(f-\sqrt{{f}^{2}+{\left(r-{r}_{0}\right)}^{2}}\right)\right)+\frac{l\cdot \theta }{2}.$$

Figure 1b shows the local arrangement of the metasurface. The distance between the adjacent ring is 330 nm, and the distance between each nanopost in the ring is 330 nm, too. The toroidal arrangement of the metasurface could make the light field of Q-PV more even. Figure 1c shows the structure of a single nanopost with a height of *H* = 600 nm, the long axis is *L* = 250 nm, and the short axis is *W* = 95 nm. The TiO_2_ nanopost is an artificial birefringent material with high refractive index at visible light wavelength. It can accurately control the emission phase delay of the partially transmitted light within 2*π* range^32^. For high permittivity metasurfaces, due to a strong local waveguide effect, the interaction among adjacent nanoposts can be ignored^32^. At the same time, the TiO_2_ nanopost has a transmittance of up to 90% in the visible light band^37^. Figure 1d shows the transmission efficiency and the phase delay of a single nanopost in the case of RCP source. It shows that the TiO_2_ nanopost has a transmission efficiency at different rotation angles is close to 1, and only if the transmission efficiency is decreased at angles of 0 or 90 degrees, still remaining above 75%. The red line with dots shows the phase delay of the TiO_2_ nanopost varying with rotation angle, which conforms to the phase-angle conversion relationship.

### The generated Q-PVs by metasurfaces

**Figure 2:**
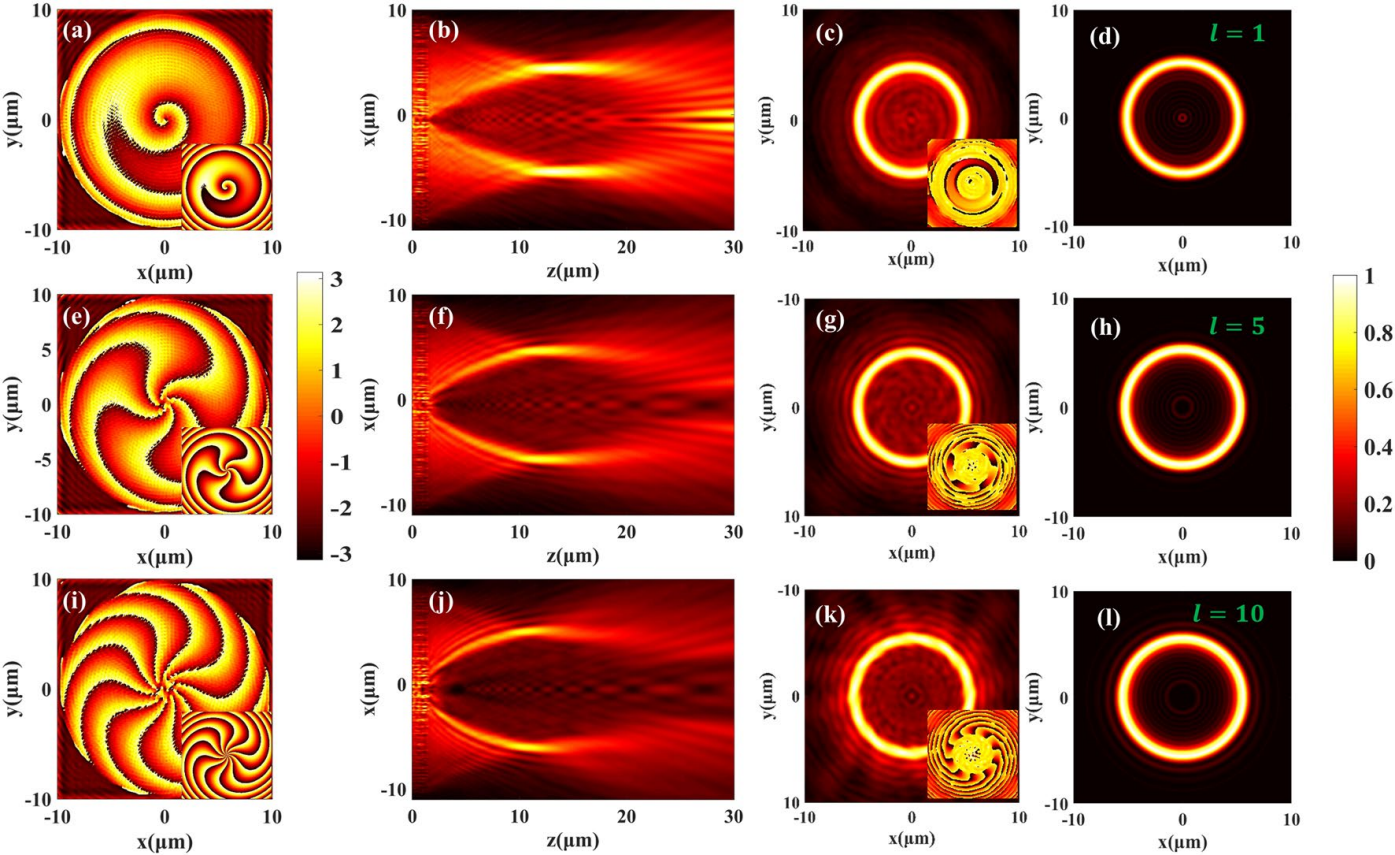
The phase and electric field distributions of Q-PVs. (**a**,**e**,**i**) The simulated phase distribution after the metasurface when *l* equals 1, 5, and 10, respectively. The insets show the designed phase distributions. (**b**,**f**,**j**) The longitudinal section of the Q-PV light field on the *xOz* plane; (**c**,**g**,**k**) The simulated electric field intensity distributions in the focal planes and the insets represent the corresponding phase distributions; (**d**,**h**,**l**) The theoretical light field calculated by Fresnel integration diffraction theory. All the simulated light field distributions were normalized.

To generate Q-PV, we firstly chose metasurfaces with aperture diameter *D* of 20 μm, *l* of 1, 5, 10, *f* of 15 μm, *r*_0_ of 5 μm to perform numerical simulations and made a comparison with the light field calculated by Fresnel integral diffraction theory. Figure 2a,e,i show the simulation results of the designed phase distributions of different TCs when *l* equals 1, 5, and 10, respectively. Phase values were obtained by extracting the transmission electrical field components and calculating with $arctan2[Im(Ex)/Re(Ex)]$. The simulated phase distributions agree with the theoretical ones. This proves that the designed metasurface can well accomplish phase control. Figure 2b,f,j respectively show the light field distributions of the Q-PV field in the *xOz* plane when *l* equals 1, 5, and 10. We can see that as the TC increases, the actual focal ring gradually deviates from the position of the designed focal length of *f* = 15 μm, which can be attributed to the Poynting vector’s oblique nature of the light field. However, the ring-shaped distribution in the designed focal plane remains unchanged. Figure 2c,g,k show the simulated light field in the focal plane with different *l*, their ring-shape distribution hardly changed with TC. The insets of Fig. 2c,g,k are the phase distribution of the corresponding focal planes. The number of the mutation in the phase pattern corresponds to the number of TC. Figure 2d,h,i show the theoretical light field distributions of Q-PVs calculated with Eq. 3, we can see that the simulation results were consistent with theoretical results. Therefore, the designed PB phase metasurface can successfully generate Q-PVs whose ring-shaped light field distributions hardly changed with TC.

**Figure 3:**
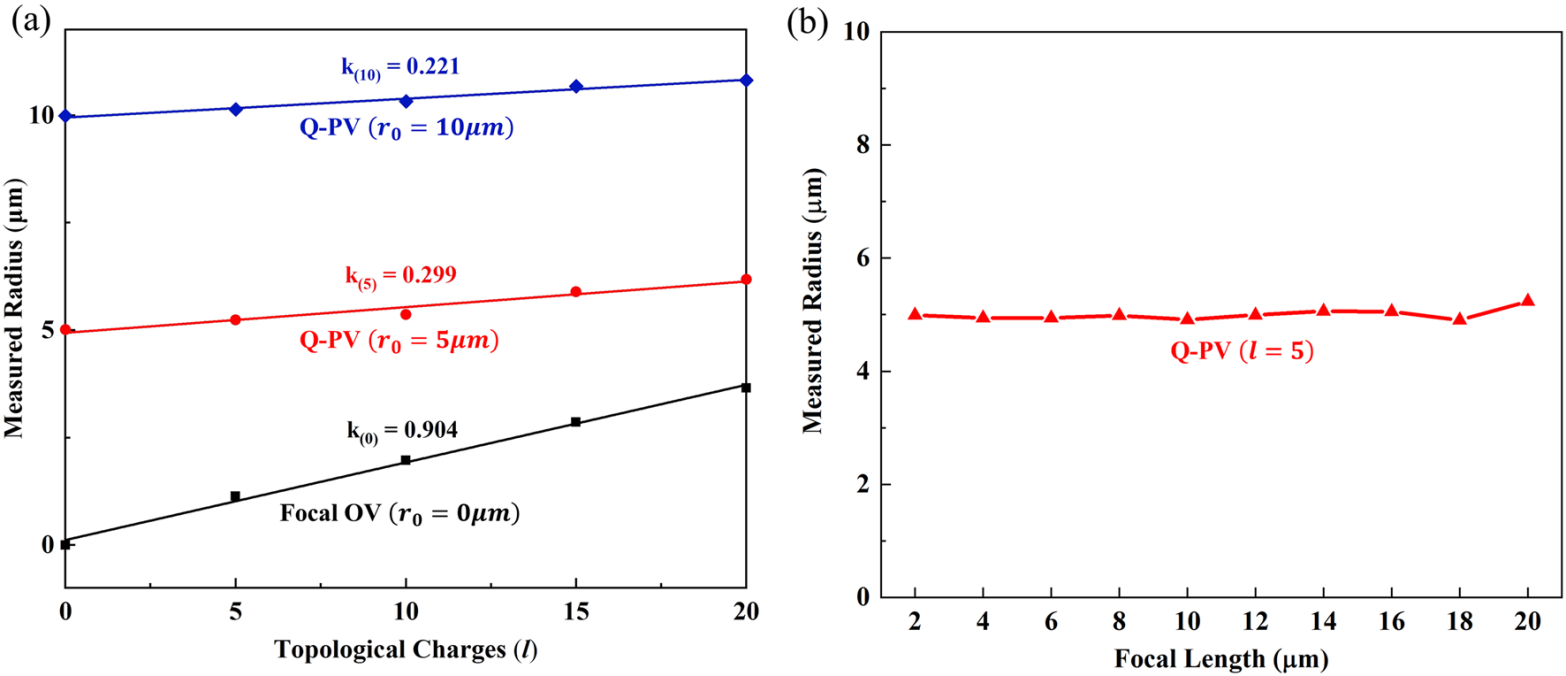
The radii of the Q-PV ring under different TCs and focal lengths. (**a**) The relationship between TC (*l* = 0, 5, 10, 15, 20) and *r*. The dots were fitted by straight-line fit method. The slopes of fitting lines with TCs of 1,5 and 10 are k_(0)_, k_(5)_ and k_(10)_, respectively. (**b**) The relationship between *f* and *r* when *l* equals to 5, *r*_0_ equals to 5 μm.

As we know, the radius of the focal OV ring is positively correlated with TC. In order to study the divergence of the Q-PV, we quantitatively calculated the relationship between the measured radius (*r*) and TC by replacing the lens phase with the annular focusing phase. When designed radii (*r*_0_) were different, as shown in Fig. 3a. We took focal OV as a comparison, as shown by the black line, the slope of the fitted line is 0.904. When *r*_0_ increases to 5 μm, we found that *r* still increased with TC, but the trend of the increase significantly reduced, appearing as the slope of the fitted straight line decreases to 0.299. When *r*_0_ was set to 10 μm, the increasing slope of the *r* was further limited to 0.221. This kind of divergence was also found in reference^33^ as the Q-PV has a lower slope for the relationship between TC and *r* when *r*_0_ was larger. Meanwhile, when the focal length was set from 2 to 20 μm, we can see that *r* hardly changes with different focal lengths, as shown in Fig. 3b. Therefore, the divergence characteristics of the Q-PV are not affected by the designed focal length.

### Optical spanners with Q-PVs

**Figure 4:**
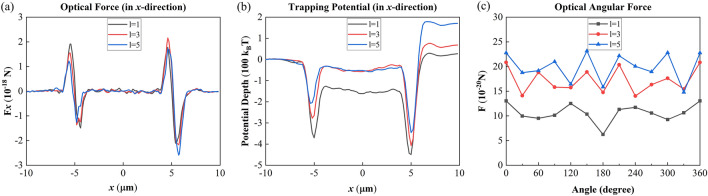
The force distributions when *l* equals 1, 3, 5. (**a**) The optical force along the *x*-direction; (**b**) The trapping potential along the *x*-direction; (**c**) The angular optical force of metallic particle every 30 degrees along the focal ring of Q-PV.

Particles can be trapped and rotated in the focal OV. Since Q-PV has fixed radius of the focal ring, we will explore the new possibility of Q-PV being used in the applications of optical tweezers and spanners. We firstly simulated the Q-PV to trap SiO_2_ Mie-particles by choosing metasurfaces with aperture *D* of 20 μm, *l* of 1, 3, 5, *f* of 15 μm, *r*_0_ of 5 μm. Meanwhile, the intensity of the incident light was set as 300 *hν* (i.e., the power of the light source was set to 0.00093 *mW*). The position of the SiO_2_ dielectric particle was set to the maximum intensity point of the focal ring (*z* = 14 μm), and along the *x*-axis from − 10 μm to 10 μm, every 0.2 μm a step. We calculated the horizontal component of optical force exerted on the SiO_2_ particles, as shown in Fig. 4a. Since the force distribution has symmetry at *x* = 5 μm and − 5 μm, *x* =  − 5 μm was taken as an example. The force at *x* =  − 6 μm shows that when the dielectric particle was close to the outer side of the focal ring, it firstly received an increasing optical force pointing to the center of the focal ring, and then the optical force gradually decreased to 0, which was just at the center of the focal ring of Q-PV (i.e., *x* =  − 5 μm). As the particle continue to move towards the inner side of the focal ring, it was pulled by increasing and decreasing optical pulling forces in turn, suggesting that the dielectric particle would be trapped in the center of the Q-PV focal ring. By using Eq. 11, we calculated the trapping potential for SiO_2_ particle that integrated from *x* =  − 10 μm, as shown in Fig. 4b. The trapping potential under each TC reached the lowest point of potential energy when the radius equals 5, and the depth of the potential wells reach below − 200 *k*_B_*T*. In general, when the depth of trapping potential reaches − 1 *k*_B_*T*, the particles can be stably trapped in the light field. Therefore, the dielectric particles can be stably trapped in the center of the orbit of the Q-PV.

Then we detected the OAM of Q-PV by detecting the angular optical force of metallic particles. The using of metallic particles is because they have high absorption and scattering that maximizes the transfer of OAM from the Q-PV^38^. Using Ag particles with a radius of 0.2 μm, we tested the angular force of the micro-sized particles every 30*°* on the focal ring, and the obtained results are shown in Fig. 4c. As TC increases, the angular optical force exerts on the metallic particles gradually increases. The fluctuation of the force distribution was due to the uneven intensity of the light field, which was caused by the discrete distribution of nanoposts of the metasurface. We concluded that Q-PV not only can stably trap particles on fixed orbits, but also it can provide optical angular force that increases with TC for optical spanner use.

### Q-PV arrays

Recently years, multichannel WDM technology based on metasurface has been favored by many researchers^39,40^. In order to design a single metasurface with multi-channel OAM states, coaxial and non-coaxial Q-PV arrays are generated by stacking the complex amplitudes of Q-PV that carrying different TCs. By superimposing complex amplitudes of different single Q-PVs, the summation complex function is calculated, and the phase profile of the Q-PV array is then determined.

By introducing a linear phase gradient along the *θ* direction onto the phase of Q-PV: $\mathrm{exp}\left[ikrS\left(\mathrm{sin}\theta \cdot \mathrm{sin}{\theta }_{n}+\mathrm{cos}\theta \cdot \mathrm{cos}{\theta }_{n}\right)/f\right]$^33^, where ($r,\theta$) is the polar coordinate parameter of each sampling point on the metasurfaces, (*S, θ*_*n*_) is the designed position of the Q-PV in polar coordinates in the focal plane, the Q-PV will propagate away from the $z$-direction at a certain diffraction angle. The single phase of Q-PV in the array could be expressed as.

6 $${\Phi }_{n}=krS\left(\mathrm{sin}\theta \cdot \mathrm{sin}{\theta }_{n}+\mathrm{cos}\theta \cdot \mathrm{cos}{\theta }_{n}\right)/f+\left(k\left(f-\sqrt{{f}^{2}+{\left(r-{r}_{0}\right)}^{2}}\right)+{l}_{n}\cdot \theta \right).$$

where *n* = 1, 2, 3, 4. By using the phase packing formula, we finally got the combinate phase of the non-coaxial Q-PV array^41^:

7 $${\Phi }_{array}=\mathrm{arg}\left\{\sum_{\mathrm{i}=1}^{n}exp\left(i{\Phi }_{\mathrm{i}}\right)\right\}.$$

Note that the italic *i* in the formula is the imaginary unit.

**Figure 5:**
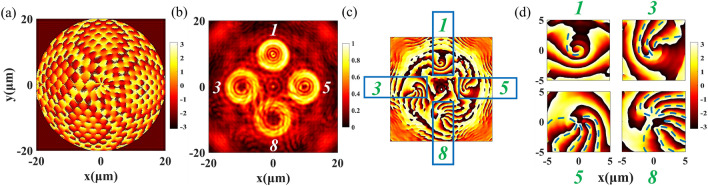
The non-coaxial array of Q-PV. (**a**) The design phase distribution of the Q-PV non-coaxial array; (**b**) The light field intensity distribution in the focal plane; (**c**) The phase distribution in the focal plane, the blue rectangle boxes out the phase of Q-PV at that position and TC; (**d**) The partially enlarged phase distributions in the focal plane, dotted lines indicate the position and number of phase fringes.

As shown in Fig. 5, a non-coaxial Q-PV array composed of four single Q-PVs is demonstrated here. The size of metalens is 40 μm × 40 μm, focal length *f* = 50 μm, the displacement distance *S*_*n*_ = 10 μm, the parameters of every single Q-PV are listed as follows, Q-PV_1_ (*l*_1_ = 1, *θ*_1_ = 90*°*), Q-PV_2_ (*l*_2_ = 3, *θ*_2_ = 180*°*), Q-PV_3_ (*l*_3_ = 5, *θ*_3_ = 0*°*) and Q-PV_4_ (*l*_4_ = 8, *θ*_4_ = 270*°*). Figure 5a shows the PB phase distribution that produced the Q-PV array. The intensity distribution of the Q-PV array at the propagation distance *z* = 50 μm is shown in Fig. 5b. The results show that the focal ring of four different TCs have the same radius. The center positions of the four Q-PVs are located at (10 μm, 90*°*), (10 μm, 180*°*), (10 μm, 0*°*), and (10 μm, 270*°*), respectively, expressed in polar coordinates, and are consistent with the designed position displacement (*S, θ*) of each Q-PV. The phase distribution in the focal plane is shown in Fig. 5c,d. The blue rectangles box out the position of each Q-PV in Fig. 5c, Fig. 5d shows the partially enlarged phase pattern. The interference fork fringe pattern can be observed near the Q-PV center, which is due to the fact that Q-PV exudes oblique upward at a certain angle after adding the linear phase gradient. The effect characteristics similar to isoclinic interference occur. The number of fork fringe reflects the TC of each Q-PV, which is consistent with the designed TC. The generated non-coaxial PV array has potential applications in the field of optical communication.

When several concentric rings that carrying different OAM information are combined to form a single metasurface array, the position of each OAM information will become certain, which is because the radius of the focal ring can maintain the designed ring radius ${r}_{0}$, especially when TC is in the range of 1 to 10. By combining Eqs. 2 & 7, the phase formula of the coaxial Q-PV metasurface array is obtained, following as.

8 $${\Phi }_{coaxial}=\mathrm{arg}\left(\sum_{\mathrm{i}=1}^{n}exp\left(ik\left(f-\sqrt{{f}^{2}+{\left(r-{r}_{\mathrm{i}}\right)}^{2}}\right)+{l}_{\mathrm{i}}\cdot \theta \right)\right).$$

**Figure 6:**
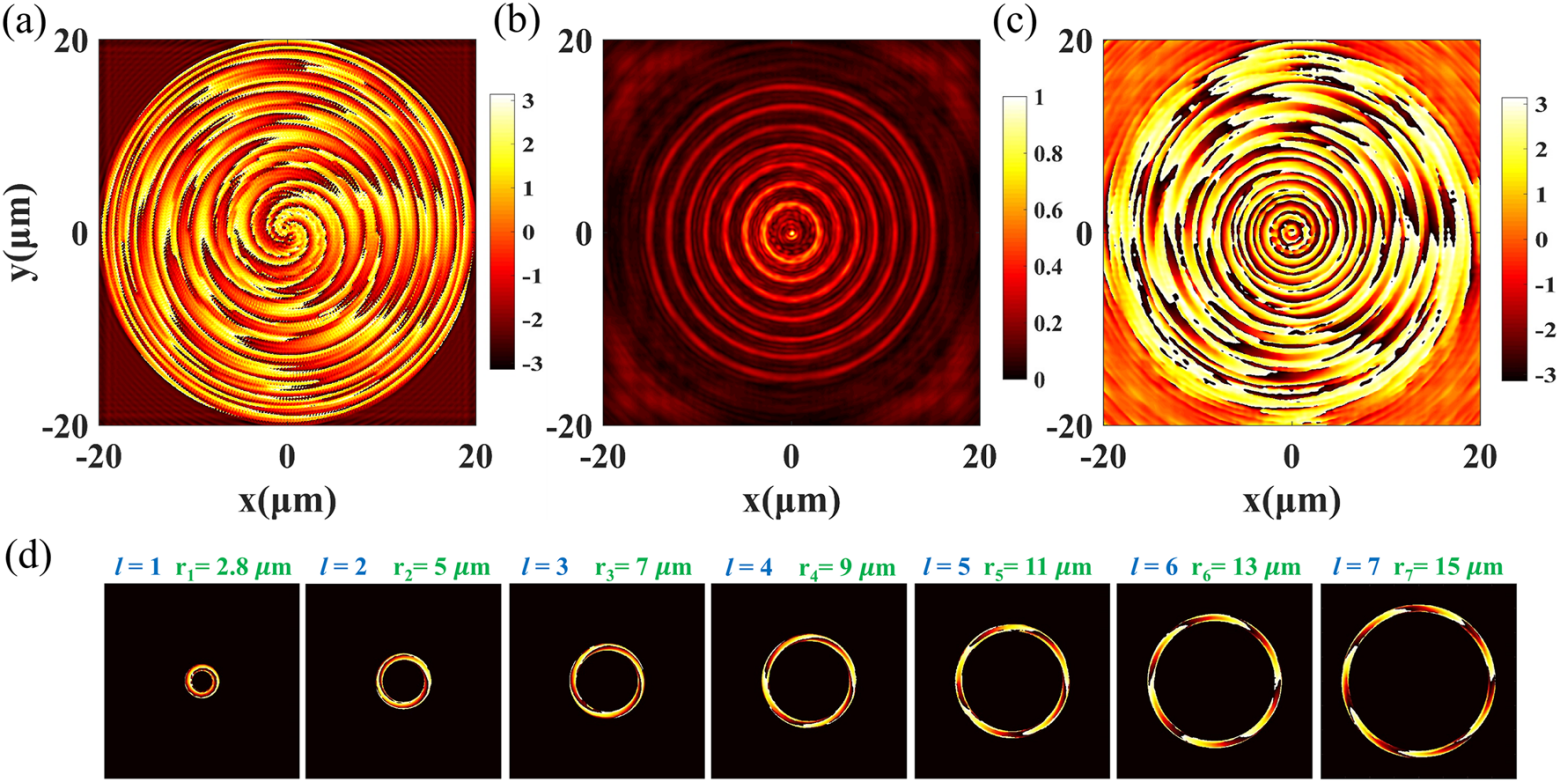
The coaxial array of Q-PV. (**a**) The designed phase distribution of the coaxial array; (**b**) The light field distribution in the focal plane; (**c**) The phase distributions in the focal plane; (**d**) The phase information of each Q-PV ring.

As is shown in Fig. 6, the size of metalens is 40 μm × 40 μm, focal length *f* = 15 μm, Δ*r* = 2 μm, *n* = 7, the parameters of every single Q-PV are listed as follows, Q-PV_1_ (*r*_1_ = 3 μm, *l*_1_ = 1), Q-PV_2_ (*r*_2_ = 5 μm, *l*_2_ = 2), Q-PV_3_ (*r*_3_ = 7 μm, *l*_3_ = 3), Q-PV_4_ (*r*_4_ = 9 μm, *l*_4_ = 4), Q-PV_5_ (*r*_5_ = 11 μm, *l*_5_ = 5), Q-PV_6_ (*r*_6_ = 13 μm, *l*_6_ = 6), Q-PV_7_ (*r*_7_ = 15 μm, *l*_7_ = 7). The phase distribution of the 7-ring coaxial array is shown in Fig. 6a. The intensity distribution of Q-PV at *z* = 15 μm is shown in Fig. 6b, and the measured radii of each Q-PV ring are *r*_1_' = 2.8 μm, *r*_2_' = 5 μm, *r*_3_' = 7 μm, *r*_4_' = 9 μm, *r*_5_' = 11 μm, *r*_6_' = 13 μm, *r*_7_' = 15 μm. They are consistent with the designed radii. The corresponding phase distribution is shown in Fig. 6c. In this case, the phase distributions at the position of each ring are less recognizable. Figure 6d shows the extraction of the phase information of each Q-PV ring, and the OAM information of each ring can be clearly distinguished, which is consistent with the number of TCs at the designed position of each Q-PV ring. The generated coaxial PV array has potential applications in the fields of ultra-high-density information storage and photon computing.

## Conclusions

In summary, as an alternative candidate to PV, our Q-PV was produced based on PB phase metasurface, it can be regarded as an annular light field with vortex phase gradient. Compared with the PVs mentioned by the predecessors, the Q-PV was generated by using a metasurface with combined phases, which can more accurately and conveniently define the focal light field. With the increase of the designed radius, the Q-PV can better get rid of the influence of TC on the radius of the maximum light intensity ring, which is not affected by focal length as well. Through the calculation of optical forces, particles can be trapped on the focal plane and rotated around the Q-PV ring. The Q-PV can be used for optical spanners that provide different angular optical forces with different TCs. Non-coaxial and coaxial arrays with Q-PVs were successfully generated, demonstrating the possibilities in the applications of optical information coding, photonic computing, and ultra-high-density information storage.

## Methods

### Simulation method

The full-wave simulation results shown in Figs. 2, 5, and 6 were performed using Lumerical FDTD solutions. The optical forces were calculated by the optical force calculation toolbox based on Maxwell stress tensor (MST) available in commercial software Lumerical FDTD solutions.

### Calculation methods

The force of micro-/nano- sized particles in the light field can be calculated by integrating the MST on the particle surface. The time-average force (including gradient force and scattering force) exerted on the particle can be expressed as

9 $$\langle F\rangle =\int \left\{\frac{\varepsilon }{2}Re\left[E\cdot n\right]{E}^{*}-\frac{\varepsilon }{4}\left(E\cdot {E}^{*}\right)n+\frac{\mu }{2}Re\left[\mu \left(H\cdot n\right){H}^{*}\right]-\frac{\mu }{4}\left(H\cdot {H}^{*}\right)n\right\}ds,$$

where $\varepsilon$ and $\mu$ are the relative permittivity and relative permeability of the medium among the particle under test, and $n$ is the normal unit perpendicular to the integration area $ds$. For further calculation of the trapping potential for a particle at *ρ*, it is necessary to integrate the optical force of particle at position from ∞ to *ρ*_0_. The trapping potential can be calculated with integral of continuous or discretized forces as follows.

10 $$\mathrm{U}\left({\uprho }_{0}\right)=-{\int }_{\infty }^{{\uprho }_{0}}F\left(\rho \right)d\rho .$$

11 $$U\left({\rho }_{n}\right)=\sum_{j=1}^{n}F\left({\rho }_{j}\right)\Delta \rho ,$$

where *ρ*_n_ is the position of particle *n* on the *x*-axis in the focal plane, Δ*ρ* is the distance between adjacent measurement points (particle *n* and particle *n* + 1), and *F*(*ρ*_*j*_) is the force on the particle at the position *j*. Generally, the trapping potential of the optical trap can usually be unified in the units of *k*_B_*T*, where *k*_B_ is Boltzmann’s constant, and *T* is normal temperature.

## Data Availability

The data that support the findings of this study are available within the paper.
